# Trimetallic Nanoalloy of NiFeCo Embedded in Phosphidated Nitrogen Doped Carbon Catalyst for Efficient Electro-Oxidation of Kraft Lignin

**DOI:** 10.3390/polym14183781

**Published:** 2022-09-09

**Authors:** Ana Maria Borges Honorato, Mohmmad Khalid, Antonio Aprigio da Silva Curvelo, Hamilton Varela, Samaneh Shahgaldi

**Affiliations:** 1 São Carlos Institute of Chemistry, University of São Paulo, São Carlos 13560-970, SP, Brazil; 2 Institut d’Innovations en Écomatériaux, Écoproduits et Écoénergies, Université du Québec à Trois-Rivières, 3351 Boul. des Forges, Trois-Rivières, QC G8Z 4M3, Canada

**Keywords:** non noble metals, doped carbon, lignin electro-oxidation, oxygen evolution reaction, hydrogen generation

## Abstract

Recently, electro-oxidation of kraft lignin has been reported as a prominent electrochemical reaction to generate hydrogen at lower overpotential in alkaline water electrolysis. However, this reaction is highly limited by the low performance of existing electrocatalysts. Herein, we report a novel yet effective catalyst that comprises nonprecious trimetallic (Ni, Fe, and Co) nanoalloy as a core in a phosphidated nitrogen-doped carbon shell (referred to as sample P-NiFeCo/NC) for efficient electro-oxidation of kraft lignin at different temperatures in alkaline medium. The as-synthesized catalyst electro-oxidizes lignin only at 0.2 V versus Hg/HgO, which is almost three times less positive potential than in the conventional oxygen evolution reaction (0.59 V versus Hg/HgO) at 6.4 mA/cm^2^ in 1 M KOH. The catalyst demonstrates a turnover frequency (TOF) three to five times greater in lignin containing 1 M KOH than that of pure 1 M KOH. More importantly, the catalyst P-NiFeCo/NC shows theoretical hydrogen production of about 0.37 μmoles/min in the presence of lignin, much higher than that in pure 1 M KOH (0.0078 μ moles/min). Thus, this work verifies the benefit of the NiFeCo nanoalloy incorporated in carbon matrix, providing the way to realize a highly active catalyst for the electro-oxidation of kraft lignin.

## 1. Introduction

Addressing the issues of environmental pollution associated with the consumption of fossil fuel has resulted in worldwide attention to the search for renewable and clean energy alternatives. Hydrogen has been touted as the ultimate power source for transportation systems and as a strategical feedstock for the industrial processes of ammonia, methanol, and heavy industries [1]. Electrocatalytic water splitting is the way forward to produce clean hydrogen when renewable electricity is utilized [2]. However, there are several challenges, including high cost, stability and the sluggish kinetics of the anodic half reaction that need to be addressed, which are the major obstacles to industrializing any water electrolyzer [3,4,5]. One approach that has been recently employed to reduce the consumption of energy for the water electrolyzer is the reforming of biomass and oxidizable organic species, which are kinetically and/or thermodynamically more favorable than the oxidation of water. During anodic oxidation, these species readily donate electrons, which react with water to produce hydrogen, resulting in lower potential than the ones required for traditional water electrolysis [4,5,6].

For instance, lignin—a source of a high content of aromatic compounds—was implemented in water electrolysis to facilitate hydrogen production, with the proposed reactions as follows [7,8]:

At cathode, the following occurs:

4H_2_O + 4e^−^ → 4OH^−^ + 2H_2_ (1)

At anode, the following occurs:

Lignin + 4OH^−^ → [O-Lignin-O] + 2H_2_O + 4e^−^ (2)

The overall reaction takes place as follows:

Lignin + 2H_2_O → [O-Lignin-O] + 2H_2_ (3)

where only a 0.2 V minimum theoretical voltage was required for the hydrogen generation in lignin assisted electrolysis, which is nearly six times lower than the theoretical voltage required for water electrolysis at 25 °C (1.23 V). Caravaca and coworkers developed a bimetallic Pt/Ru catalyst impregnated in a micro-porous layer and employed it for the electrolysis of lignin to generate hydrogen at 80 °C [9]. However, they found lower potential for the electro-oxidation of lignin than the standard potential of water decomposition (~1.23 V), the current density was much lower for the hydrogen production rate (0.4 μmol/s) [10]. In another study [11], a Pt/Ru catalyst was used for the electro-oxidation of 2-phenoxyethanol, a molecule that models β-O-4 linkage in lignin. Despite showing a promising lower potential value (<1.2 V) for the electro-oxidation of 2-phenoxyethanol at 90 °C, it suffers from poor selectivity and detachment of the catalyst during the reaction process.

The nickel-based catalysts were also exploited for lignin electro-oxidation using an ion exchange membrane electrolyzer at 80 °C to produce hydrogen [12]. Here, the selectivity of Ni/C to cleave the linkage of β-O-4 was emphasized, explaining the enhanced amount of hydrogen production. Similarly, the lignin was electro-oxidized by utilizing Ni/NiOOH, Pt, and graphite as catalysts in 1 M NaOH (pH 14, 20 g_Lig_/L) [13]. Among them, the Ni/NiOOH catalyst showed better performance by achieving a current density peak of 2 mA cm^−2^ at 0.35 V. Mahtab and coworkers [14] reported that the lignin electro-oxidation on nickel and cobalt incorporated TiO_2_ nanoparticles in an anion exchange membrane electrolyzer for the hydrogen production and found thermodynamically more favorable electro-oxidation of lignin (1.4–1.5 V) than OER. Zou and coworkers [15] used Ti/PbO_2_ for electro-oxidation of lignin and demonstrated that the overall cell voltage could be reduced by 546 mV in the presence of lignin compared to water electrolysis. In another investigation, it has been shown that the production of hydrogen rate can be tuned by variation in the concentration of lignin and temperature [15]. Duan and coworkers [16] reported the selectivity of a MnCoOOH/NF catalyst toward the electrochemical oxidation of a 1-phenylethanol molecule and revealed that high-valence metallic atoms modulate the electronic structure of CoOOH, resulting in less energy consumption to overcome the activation barrier for the electro-oxidation of 1-phenylethanol.

Several other works in the literature have also clearly evidenced the substantial progress on the reformation of biomass and alcohols for the value-added chemicals and hydrogen generation [17,18,19,20,21,22,23,24]. Finding cheap and effective electrocatalyst for the electro-oxidation of kraft lignin in practical applications is promising but challenging. Herein, a simple yet effective approach has been explored for the synthesis of an efficient electrocatalyst based on NiFeCo nanoalloy embedded in heteroatom-doped carbon and successfully employed for the electro-oxidation of kraft lignin under various operational conditions, combining with theoretical calculation of hydrogen production. The novelty of this work consists of the fact that an effective nonprecious ternary nanoalloy of NiFeCo embedded in phosphidated nitrogen doped carbon is synthesized by a simple straightforward chemical mixing method, followed by a carbonization step. The electro-oxidation activity toward lignin of the as-synthesized electrocatalyst system, therefore, does not rely on precious metal or a complex synthetic route, as previously reported for precious and nonprecious metal electrocatalysts [9,10,11,12,13,14,15,16].

## 2. Experimental Details

### 2.1. Chemical and Reagents

Kraft lignin was generously provided by ‘Suzano Papel e Celulose’ a Brazilian pulp and paper company. The lignin was obtained by acidification of black liquor from kraft pulping of Eucalyptus urograndis wood. All chemicals used in this study were from Sigma Aldrich (St. Louis, MO, USA).

### 2.2. Synthesis of P-NiFeCo/NC Catalyst

The catalyst was synthesized according to our previously reported work with some modifications [25]. Briefly, 1 mole of each metallic salt was added to 15 mL of a mixture of water and methanol (1:3) containing 2-methylimidazole and 2,6-naphtalene dicarboxylic acid dipotassium with ratio of 5:1. The mixture was left under stirring overnight at 50 °C. The resulting materials were collected, filtered, and dried in an oven at 70 °C. Thereafter, the material was carbonized at 900 °C, in a device which consisted of a tubular furnace under argon gas flow (75 mL/min) with a 2 °C/min ramp rate followed by phosphidation treatment at 200 °C for 2 h by placing sodium hypophosphite monohydrate (H_2_NaO_2_P.H_2_O) in one extremity of ceramic boat and the product in the opposite extremity. The control sample was synthesized by a similar method but without phosphidation, and was referred to as sample NiFeCo/NC.

### 2.3. Characterization

The morphological analysis of the material was performed using a Thermo Scientific Talos, 200X equip with high-angle annular dark-field (HADF-TEM) to obtain transmission electron microscopy (TEM) images with an accelerating voltage of 300 kV. For this, the catalyst was dispersed in ethanol and a single drop was deposited on a carbon-film coated copper grid (EMS, 400 mesh). The XRD data was collected using a Bruker D8-advanced diffractometer equipped with a Cu Ka radiation source (λ = 1.54 °A) between 10 to 100 degrees with a step of 0.05 °/s. Raman spectra were acquired using a LabRam, HR UV–visible–NIR, system (200–2100 nm) by employing 532-nm laser excitation. Then, X-ray photoelectron spectroscopy (XPS) experimental data was acquired by using a PHI-5300, ESCA spectrometer (PerkinElmer), employing monochromatic Al-Ka X-ray beam.

Cyclic voltammetry (CV) and linear sweep voltammetry (LSV) experiments were performed using a Metrohm-autolab (the Netherlands) workstation operated by NOVA 2.1.3 software. The reference, counter, and working electrodes were Hg/HgO, a standard graphite rod (Alfa-Aesar, (Haverhill, MA, USA) 99.999%), and a stationary glassy carbon, respectively. For the OER analysis, 1 M KOH solution was used. For the electro-oxidation of lignin, 1.0 M KOH + 10 gL^−1^ of lignin (named as *e.c.l.* throughout the text) solution was used. The LSV tests were performed at scan rates of 10, 20, 30, 40, 50, and 60 mV/s consecutively. The CV was conducted 8 to 10 times at each scan rate before analyzing LSV curves. All electrochemical experiments were conducted in a closed cell containing either 50 mL of 1 M KOH or 1 M KOH containing 10 g/L lignin kraft (e.c.l.), under a continuous purge of argon gas. The ink of the material was prepared by mixing 2 mg of catalyst powder, 40µL of Nafion (5 wt%), and 400 µL of ethanol using a sonication bath for 15 min. Then, 20 µL of ink was dropped onto the glassy carbon electrode mounting with an area of 0.2 cm^2^, with a loading of ~0.5 mg/cm^2^ of the catalyst.

## 3. Results and Discussion

### 3.1. Synthesis of Catalyst

**Figure 1:**
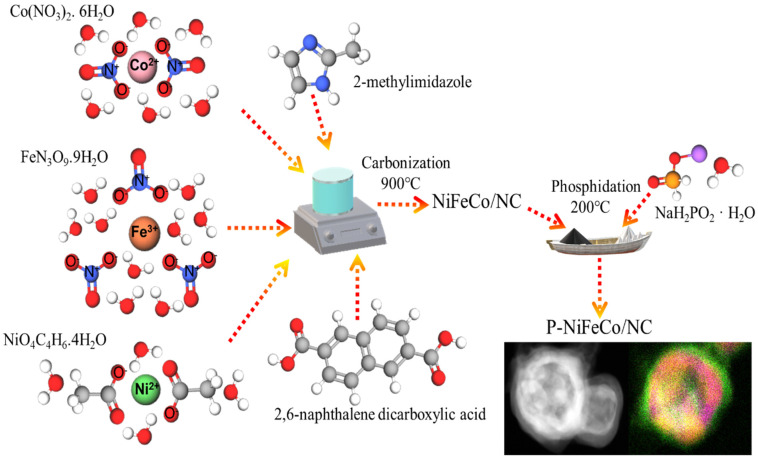
Synthesis procedure of the P-NiFeCo/NC catalyst.

For the preparation of the catalyst material, firstly an equimolar amount of Ni, Fe, and Co salts were dispersed in the water and methanol solution containing 2-methylimidazole and 2,6-naphthalenedicarboxylic acid, as illustrated in Figure 1. During the reaction process, the metal ions coordinated with electron-donating organic ligands to form the metal-organic framework assembly [25]. After carbonization of the material at 900 °C, the material was processed for phosphidation using a phosphidating agent (H_2_NaO_2_P.H_2_O) at 200 °C for 2 h in a ceramic boat under continuous argon flow (75 mL/min), which resulted in the trimetallic nanoalloy becoming embedded in the carbon catalyst.

### 3.2. Physicochemical Characterizations

**Figure 2:**
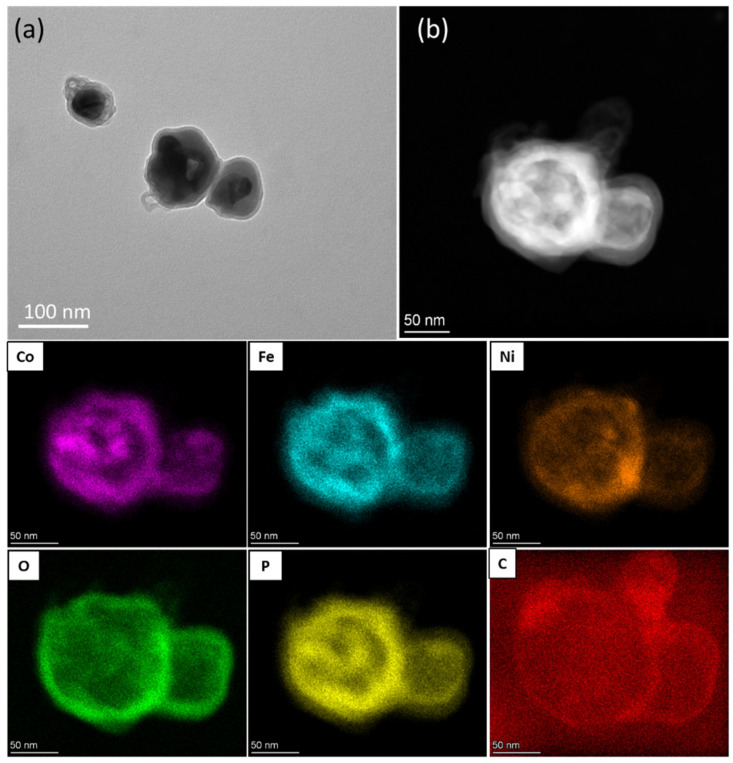
(**a**) TEM image, (**b**) HAADF image, and its corresponding EDX mapping of the Co, Fe, Ni, P, O, and C elements.

The TEM (Figure 2) and HAADF images, and the corresponding EDX mapping (Figures S1 and S2, Supporting Information), revealed the spherical morphology of the catalyst between the range of 50 to 100 nm in size, constituting a homogeneous distribution of Ni, Fe, and Co incorporated in the carbon shell. The quantification of the EDX spectrum (Figure S3a,b Supporting Information) exhibited the elements of C (41,6%), Fe (8.8%), Ni (17.9%), Co (10%), O (9.8%), N (0.4%), and P (11.5%).

**Figure 3:**
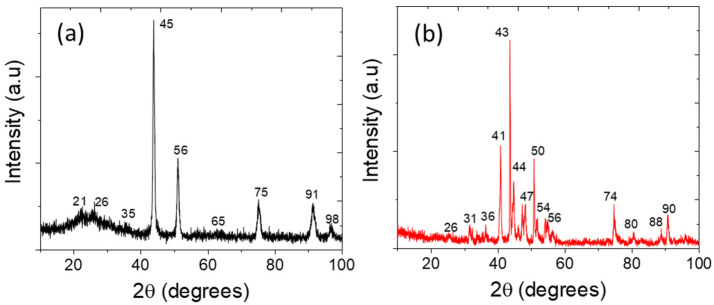
(**a**) XRD patters for NiFeCo/NC and (**b**) P-NiFeCo/NC.

The fine-tuned carbon shell comprising metallic nanoalloy is considered beneficial to improving the electrochemical performance and durability of the catalyst [25,26,27]. The intimate contact developed between the interface of carbon shell and metal core, as well as the large electro-negativity difference between both components, facilitated the charge transfer from metal core to carbon shell, resulting in high catalytic activity [26,27,28]. The X-ray diffraction pattern (XRD) of both phosphidated and non-phosphidated samples show the characteristic peaks at 2θ = 26° (Figure 3a,b) which coincided to the plan of (002) graphitic carbon [29,30]. The characteristic peaks located at 35°, 45°, 56°, 65°, 75°, 82°, 91°, and 98° denote the mixed phases of Ni, Fe, and Co for the sample of NiFeCo/NC. The peaks at 31°, 36°, 41°, 44°, 47°, 54°, 56°, 74°, 80°, 88°, and 90° for the sample P-NiFeCo/NC also represented the mixed phases of metals, as well as the phosphidated and oxidized form of the metallic alloy. The peaks at 31°, 36°, 50°, and 56° correspond to the (011), (111), (103), and (301) planes of a cobalt-containing phosphorus structure, respectively [31,32,33,34]. Some pronounced diffraction peaks for the P-NiFeCo/NC were also found at 43°, 52°, and 74° which coincided well with the (201), (300), and (212) lattice plans of P, Fe, and Ni, respectively [33,34,35,36,37,38,39], confirming the successful phosphidation of the catalyst system, which may provide more active centers for the electro-oxidation of kraft lignin originated from the structural modulation and the synergistic effect.

**Figure 4:**
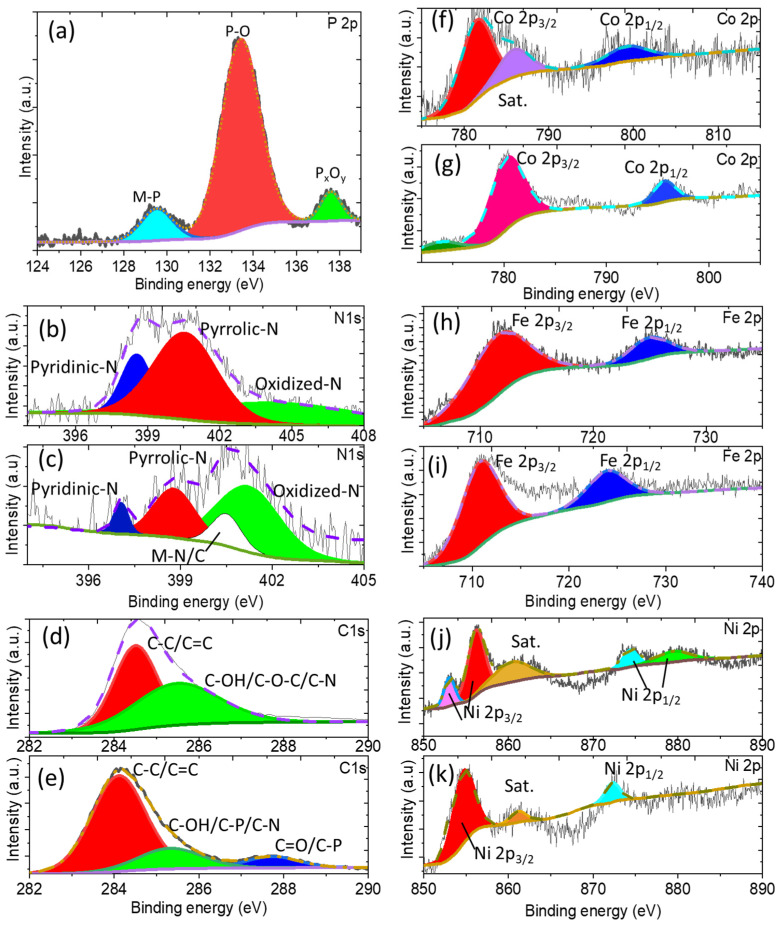
Deconvolution of high resolution XPS spectra for (**a**) phosphorus of P-NiFeCo/NC, (**b**,**c**) nitrogen of NiFeCo/NC and P-NiFeCo/NC, (**d**,**e**) carbon of NiFeCo/NC and P-NiFeCo/NC, (**f**,**g**) cobalt of P-NiFeCo/NC and NiFeCo/NC, (**h**,**i**) iron of P-NiFeCo/NC and NiFeCo/NC, and (**j**,**k**) nickel of P-NiFeCo/NC and NiFeCo/NC.

The X-ray photoelectron spectroscopy (XPS) survey showed the presence of elements in the P-NiFeCo/NC and NiFeCo/NC catalysts (Figure S4, Supporting Information). Figure 4a shows a high-resolution P 2p XPS spectrum, where peaks centered at 130.5 eV and 129.3 eV correspond to the binding energies of phosphorus and metals, assigning to 2p_3/2_ and 2p_1/2_, respectively. The broad peak at 133.3 eV can be attributed to P-O linkage or the oxidized phosphorus species, and the peak located at 138 eV may be ascribed to the P_x_O_y_ species [29,30,31,32,33]. The high-resolution N 1s spectrum of NiFeCo/NC (Figure 4b) exhibits the existence of three types of the nitrogen species pyridinic-N at 398.7 eV, pyrrolic-N at 401.1 eV, and oxide-N at 403.2 eV. In the high-resolution N 1s spectrum of the sample P-NiFeCo/NC, besides pyridinic-N (397 eV), pyrrolic-N (399 eV) and oxidized-N (401 eV), it showed additional peak for the metal–nitrogen/or phosphorous bond at 400.5 eV (Figure 4c). The coordination between metal and nitrogen-doped carbon can create a strong electron-withdrawing effect, favoring a high catalytic performance [28,29,30,31,32,33,34,35,36]. The deconvolution of carbon C1s for both samples (Figure 4d,e) showed the presence of oxygen functionalized carbon. The catalyst P-NiCoFe/NC demonstrated an additional peak at 288 eV, which may be ascribed to the phosphidated carbon, as shown in (Figure 4e) [29,34].

The high-resolution Co 2p spectrum of P-NiFeCo/NC (Figure 4f) represented three peaks. The peaks at 781.5 eV and 799.5 eV correspond to the Co-P bonds of Co 2p_3/2_ and Co 2p_1/2_, while the third peak located at 785 eV was ascribed to the oxidized and hydroxide cobalt species (Co^2+^) with a satellite of 2p_3/2_, respectively [29,30,31,32,33,34,35]. Figure 4g, shows peaks at 780.4 eV and 795.6 eV for NiFeCo/NC assigning to Co 2p_3/2_ and Co 2p_1/2_, respectively, while the peak at 775 eV represents oxidized Co^+3^ [32]. It is worth noting that, after phosphidation, the catalyst observed a small shift in the binding energy of the Co 2p_3/2_, from 780.4 eV (NiFeCo/NC) to 781.5 eV (P-NiFeCo/NC), and for the Co 2p_1/2_ from 795.6 eV (NiFeCo/NC) to 799.5 eV (P-NiFeCo/NC). These shifts suggested the changes in the coordination bonds and electronic structure of the catalyst after phosphidation [32,36,37,38,39]. Figure 4h denoted Fe 2p_3/2_ and Fe 2p_1/2_ at 712.5 eV, and 726 eV for the P-NiFeCo/NC. As for NiFeCo/NC (Figure 4i), the Fe 2p_3/2_ and Fe 2p_1/2_ were located at 711 eV and 724 eV, respectively [36,37,38,39]. The deconvolution of Ni 2p for the P-NiFeCo/NC and NiFeC/NC has been shown in Figure 4j,k, respectively. The observed binding energies at 853.1 eV and 856.2 eV were dispensed to Ni 2p _3/2_, whereas the peaks located at 875 eV and 880 eV represented for Ni 2p_1/2_, including a satellite peak at 861.2 eV for the P-NiFeCo/NC. The peaks of Ni 2p _3/2_ and Ni 2p _1/2_ for the NiFeCo/NC were attributed to the binding energies of 852.7 eV and 872.3 eV with a satellite peak at 860 eV [32,33,34,35,36]. The higher shift in binding energies of the Ni 2p_1/2_ (875 eV and 880 eV)_,_ Ni 2p_3/2_ (856.2 eV), Fe 2p_3/2_ (712.5 eV), and Fe 2p_1/2_ (726 eV) for the P-NiCoFe/NC compared to the Ni 2p_1/2_ (872.3 eV)_,_ Ni 2p_3/2_ (852.7), Fe 2p_3/2_ (711 eV), and Fe 2p_1/2_ (724 eV) peaks of NiCoFe/NC indicated the strong change in electronic structure and the local environment of Ni, Co, and Fe [32,36,37,38,39]. The high-resolution XPS spectrum of O 1s for the P-NiFeCo/NC and NiFeCo/NC (Figure S5a,b, Supporting Information) showed the existence of hydroxyl-O species at 529.6, 531.0, and 532.2 eV, which were attributed to the oxide linkages of metals [33,35,36,37,38]. In the Raman spectra of P-NiFeCo/NC and NiFeCo/NC (Figure S6, Supporting Information), the appearance of the G band at 1598 cm^−1^ confirms the existence of graphitized carbon, and the D band at 1328 cm^−1^ corresponds to disordered carbon, corroborating the XRD results [25]. The ratio of the D and G bands (I_D_/I_G_) for the NiFeCo/NC and P-NiFeCo were calculated to be 0.79 and 0.75, respectively, representing the higher structural disorder in catalyst materials [25,31].

### 3.3. Electrochemical Characterizations

**Figure 5:**
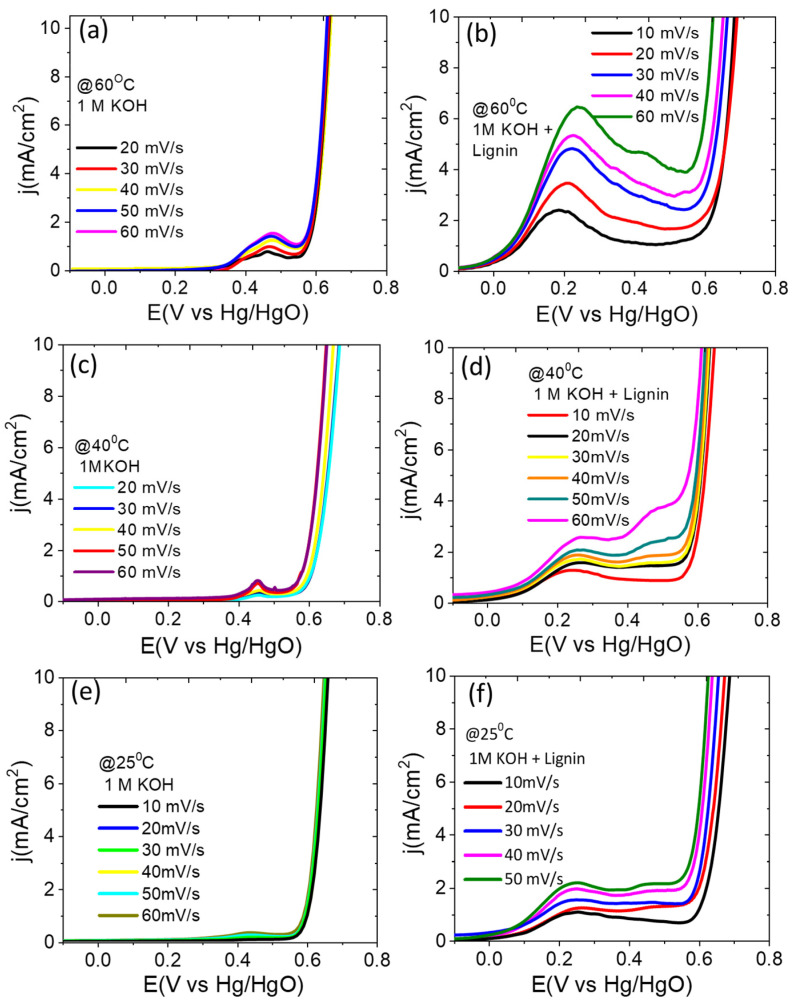
LSV curves for the P-NiFeCo/NC at different temperatures in (**a**,**c**,**e**) 1 M KOH and (**b**,**d**,**f**) in *e.c.l.* electrolyte.

**Figure 6:**
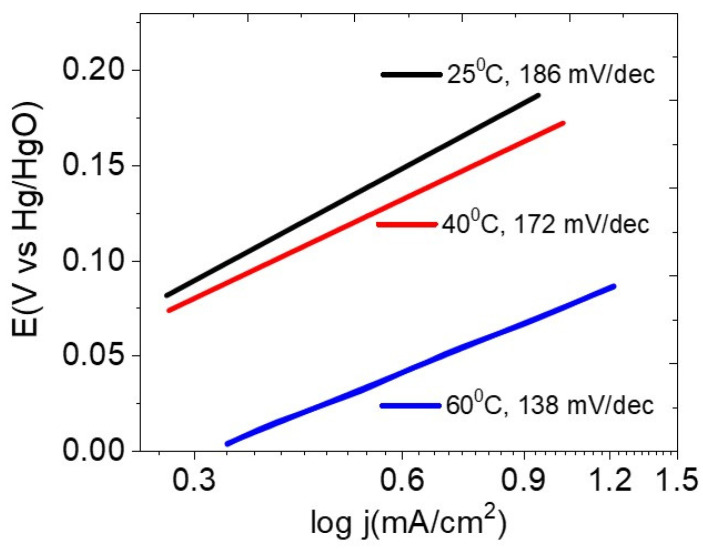
Tafel slope for the P-NiFeCo/NC at different temperatures in *e.c.l.* electrolyte.

Firstly, the electrochemical activities of the sample P-NiFeCo/NC were analyzed in 1 M KOH and *e.c.l.* (1 MKOH + 10 g/L lignin) solutions with respect to the scan rates at different temperatures. As can be seen in Figure 5, the LSV curves of P-NiFeCo/NC in 1 M KOH at 60, 40, and 25 °C show very small oxidation peaks between 0.4–0.5 V vesus Hg/HgO with increasing scan rates from 20 to 60 mV/s. Meanwhile, the same catalyst in the lignin containing electrolyte (*e.c.l.*) showed more pronounced oxidation peaks under the lower potential range from 0.15–0.3 V versus Hg/HgO than the one analyzed in 1 M KOH solution, confirming that the observed higher oxidation peaks are solely originating from the desirable lignin electro-oxidation on the catalyst surface [40,41,42]. The LSV curves of P-NiFeCo/NC in the *e.c.l.* also showed that the peak current was linearly increased as the scan rate increases, whereas, in the 1 M KOH electrolyte, the peak current was much suppressed. The oxidative peak currents were highest at 60 °C with respect to the increasing scan rates, following the order of 3.43 mA/cm^2^ @ 20mV/s, <4.8 mA/cm^2^ @ 30mV/s, <5.31 mA/cm^2^ @ 40mV/s, <5.92 mA/cm^2^ @ 50mV/s < 6.43 mA/cm^2^ @ 60mV/s. The highest peak current density of 6.43 mA/cm^2^ was achieved at 60 °C with a scan rate of 60 mV/s, suggesting the enhanced kinetics of lignin electro-oxidation increases with increasing temperature, resulting in a higher conversion efficiency of lignin [37,40]. This was also verified by observing a smaller Tafel slope value of 138 mV/dec at 60 °C compared with 40 °C (172 mV/dec) and 25 °C (186 mV/dec), representing the temperature dependent reaction kinetics of P-NiFeCo/NC for the electro-oxidation of lignin (Figure 6).

The above electrochemical results are consistent with the recent investigation of Caravaca [9], where an Ni/C electrode was implied for the electro-oxidation of phenoxyethanol (as a lignin-model) in 1 M KOH at 60 °C (20 mV/s), showing the onset potential of 0.4 V versus Hg/HgO and a current density of 10 mA/cm^2^ at 600 mV versus Hg/HgO. After the first set of oxidation peaks near to 0.2 V versus Hg/HgO, the current density gradually decreased as the potential progressed toward more positive values. This indicates that the current reached its limit due to the diffusional limitations [40,41,42,43]. This is similar to the Ni/C and Co/C electrodes, where a decrease in current was observed for the lignin oxidation due to the reduced active sites caused by the adsorbed reactant species on the electrode surface [42,44,45]. As the potential was swept toward a more positive value of 0.55 V versus Hg/HgO, a sharp increase in current took place due to the mixed electro-oxidation of water and lignin [12,41,46]. Compared to the P-NiFeCo/NC, the non-phosphidated catalyst (NiFeCo/NC) (Figure S7, Supporting Information) showed inferior activity toward lignin electro-oxidation by displaying lower oxidative peak currents at different scan rates, following the order of 3.2 mA/cm^2^ @ 20mV/s, <4.0 mA/cm^2^ @ 30 mV/s, <4.3 mA/cm^2^ @ 40 mV/s, <4.27 mA/cm^2^ @ 50 mV/s < 4.51 mA/cm^2^ at 60 mV/s in *e.c.l.* electrolyte. This result confirms the higher activity and selectivity of the P-NiFeCo/NC toward lignin electro-oxidation in alkaline solution mainly because of the unique set of combinations of the ternary nanoalloy of NiFeCo confined in doped carbon shell, which promotes the fast kinetics for lignin electro-oxidation. The standard IrO_2_/C (20 wt%) catalyst was also tested and compared with the as-synthesized P-NiFeCo /NC and NiFeCo /NC at 60 °C (@ 20 mV/s scan rate) in *e.c.l.* electrolyte. As can be seen in (Figure S8, Supporting Information), the lignin electro-oxidation peaks for the P-NiFeCo/NC and NiFeCo/NC were at lower potential of ~0.2 V versus Hg/HgO compared to the peak observed for the IrO_2_/C at 0.24 V versus Hg/HgO. However, the currents for the P-NiFeCo/NC (7.7 mA/mg) and NiFeCo/NC (6.98 mA/mg) were found slightly lower than that of the IrO_2_/C (9.2 mA/mg). The observed electro-oxidation activity of the as-prepared catalyst at low potential was fairly high compared to several lately reported precious and nonprecious electrocatalysts [9,10,11,12,13,14,15,16]. Our results also corroborate the observation of Staser and coworkers [41], where they observed ~ 3.5 mA/cm^2^ at 0.8 V versus SHE (@50 mV/s), using a β-PbO_2_ electrocatalyst with mass activity of 2.34 A g^−1^(0. 35 V versus SHE) for the lignin electro-oxidation.

Furthermore, the intrinsic trait of the catalyst was examined by the turnover frequency (TOF), which measures the number of products generated per active site per unit time [46,47,48]. Indeed, the oxidation peaks that appear during the electrochemical reaction are associated to the redox activity of the catalyst and can be utilized to calculate the surface-active sites of the catalyst [46,49,50,51]. The concentration of active sites per surface area was calculated using the slope from the curve of oxidative peaks versus scan rates, according to the following equation [49,50,51]:

(4) $$\frac{F^{2}n^{2}A\Gamma_{O}}{4RT}=slope$$

The slopes were calculated from the curves as shown in Figure S9 (Supporting Information). Thus, the TOF was calculated using the current density (*J*) at a specific potential, the number of moles of active sites (*A_S_*), and the mole of electrons participated for evolving one mole of O_2_ from water (e*), as is shown in the equation below [45,48,49,50,51]. In the traditional water splitting, e* corresponds to four electrons, which were also assumed to be four in electro-oxidation of lignin [48,49,50,51]. The equation is as follows:

(5) $$\mathrm{TOF}=\frac{JA}{e^{*}F\;A_{s}}$$

**Figure 7:**
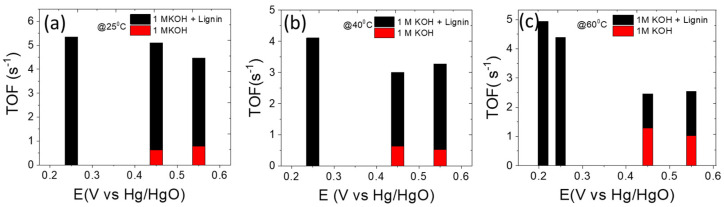
(**a**–**c**) Plots of the TOF values of P-NiFeCo/NC.

The concentration of active sites (*A_S_*) per surface area (mols/cm^2^) of P-NiFeCo/NC was calculated at different temperatures, as follows: 5.33 × 10^−10^ (25 °C), 1.2 × 10^−9^ (40 °C), and 1.81 × 10^−9^ (60 °C). Then, the TOF for the P-NiFeCo/NC catalyst at two different potentials of 0.45 and 0.55 V versus Hg/HgO were calculated to be 0.6 s^−1^ (25 °C), 0.62 s^−1^ (40 °C), 1.2 s^−1^ (60 °C) and 0.79 s^−1^ (25 °C), 0.53 s^−1^(40 °C) and 1.0 s^−1^ (60 °C), respectively in 1 M KOH solution (Figure 7a–c). When TOF was calculated for the same catalyst P-NiFeCo/NC in *e.c.l.* electrolyte, it showed much higher values compared to those observed in 1 M KOH solution. For example, at potentials of 0.25, 0.45, and 0.55V versus Hg/HgO, the TOF values were 5.3, 5.1, and 4.4 s^−1^ at 25 °C, 4.1, 3.0, and 3.2 s^−1^ at 40 °C, respectively. At 60 °C, the TOF values were calculated as being about 4.9 s^−1^ (0.21 V), 4.4 s^−1^ (0.25 V), 2.46 s^−1^ (0.45 V), and 2.5 s^−1^ (0.55 V), as shown in Figure 7c. The TOF value at the low potential 0.21 was measured due to high current density of the catalyst at 60 °C compared to 25 and 40 °C. These results revealed that the catalyst owns a remarkable intrinsic feature for the lignin electro-oxidation.

### 3.4. Theoretical Calculations for Hydrogen Production

The oxidation of lignin can also be considered for hydrogen production as it enhances the thermodynamic efficiency of the system by rapidly donating electrons to the cathode to reduce protons at the cathode to generate hydrogen molecules, resulting in an energy saving trait compared with conventional water electrolysis [9, 10, 11, 12, 15, 18]. The evolution of hydrogen can be calculated by Faraday’s equation (as shown below), where the amount of hydrogen generated ($mol\;H_{2}{\;}_{s^{-1}}$) is proportional to the current I (A) produced during the electro-oxidation of oxidizable species [15,18].

*mol H*_2_*s*^−1^ = *I*/*2F* (6)

**Figure 8:**
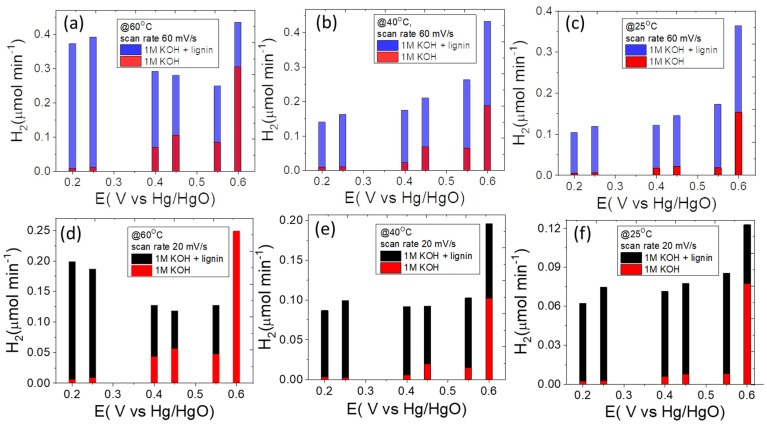
(**a**–**f**) Theoretical hydrogen production (μmol.s^−1^) in conventional water electrolysis and in lignin electro-oxidation at different temperatures and scan rates for the catalyst P-NiFeCo/NC.

**Figure 9:**
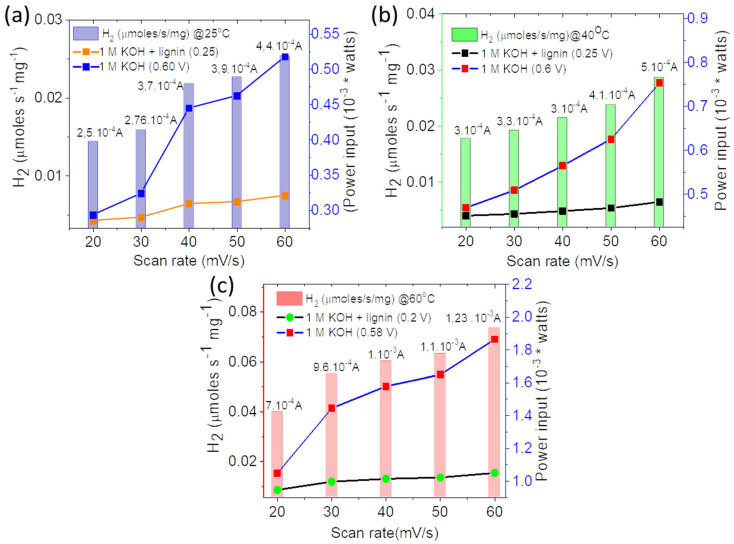
(**a**–**c**) Theoretical hydrogen generation (μ moles s^−1^ mg^−1^) in conventional water oxidation and in lignin electro-oxidation at different scan rates.

Accordingly, the estimated amount of H_2_ produced in the presence of lignin is much larger than the one produced by traditional water electrolysis at different potentials and scan rates (Figure 8a–f). Theoretically, by obtaining higher currents at lower potential during the electro-oxidation of lignin, lower cell voltages will be required to generate hydrogen. It is interesting to note that if hydrogen evolution reaction takes place near to 0 V at the cathode, then the overall cell voltage will be directly proportional to the anodic potential, meaning that the cell voltage will depend on the potential of anode at which the electro-oxidation of oxidizable species occurs [15,18]. The theoretical results calculated herein are in agreement with the oxidation of lignin that requires much lower potential for hydrogen generation than in a conventional method [7,9,10,11,12,19]. Figure 9 represent the power input required to generate hydrogen in both electrolytes by employing the equation P = IV, where V represents the voltage required to sustain the current (I). As can be observed from Figure 9a–c, the OER in 1 M KOH required almost double the power input than lignin containing electrolyte to generate the same amount of current and hydrogen.

## 4. Conclusions

In conclusion, we have elaborately fabricated a ternary metal nanoalloy of NiFeCo integrated in phosphidated nitrogen-doped carbon, facilely followed by a simple chemical method and carbonization step. The obtained electrocatalyst achieved much lower overpotential for the electro-oxidation of lignin when compared with conventional water electrolysis under various operational conditions. As evidenced by physical and electrochemical characterizations, the excellent electro-oxidation of lignin is originated from the uniquely embedded ternary metal nanoalloy of NiFeCo in a doped carbon shell, giving lignin electro-oxidation activity between a potential range of 0.2 to 0.25 V versus Hg/HgO, much lower than the conventional OER (0.59 V versus Hg/HgO). A linear dependence of current with respect of temperatures and scan rates revealed that the electro-oxidation of lignin is being controlled by mass transport. In particular, the current produced per gram of catalyst and estimated theoretical value of hydrogen production per gram of catalyst were also reported, showing that the use of lignin as an oxidizable species can reduce by two times the power input needed to generate hydrogen in comparison to conventional OER.

## Data Availability

The data used to support the findings of this research work are included within the article and Supporting Information, more data may be available from the corresponding author on reasonable request.

## Supplementary Materials

The following supporting information can be downloaded at: https://www.mdpi.com/article/10.3390/polym14183781/s1, Figure S1: HAADF-TEM image of NiFeCo nanoalloy and its corresponding elemental mappings Co, Ni, Fe, P, O, and C; Figure S2a,b: TEM image of carbon shell protected trimetallic-nanoalloy; Figure S3a: EDX mapping for the homogeneous distribution of the elements and b) scan line spectrum for the quantification of the elements C (41.6%), Ni (17.9%), P(11.5%), Co(10%), O (9.8%), Fe(8.8%) and N(0.4%); Figure S4: XPS survey of the samples P-NiFeCo/NC (in pink) and NiFeCo/NC (in blue) showing the peaks of the elements at their specific binding energies; Figure S5: O1s peaks deconvolution of the sample P-NiFeCo/NC a) and NiFeCo/NC b); Figure S6: Raman spectroscopy of the samples P-NiFeCo/NC (in red) and NiFeCo/NC (in black) showing the G and D bands; Figure S7: LSV tests of NiFeCo/NC in 1 MKOH and b) in 1 M KOH + lignin at 60 °C; Figure S8: LSV tests of IrO_2_/C, P-NiFeCo/NC, and NiFeCo/N at 60 °C, 20 mV/s in lignin electro-oxidation in 1 M KOH + lignin; Figure S9a,b,c: Curves of current at lignin oxidation peaks (mA) versus scan rates from the LSVs taken at 25 °C,40 °C and 60 °C.
