# Supplementary material for: Trimetallic Nanoalloy of NiFeCo Embedded in Phosphidated Nitrogen Doped Carbon Catalyst for Efficient Electro-Oxidation of Kraft Lignin
Source: Polymers (Basel). 2022 Sep 9;14(18):3781. doi: 10.3390/polym14183781 (PMC9503039; doi:10.3390/polym14183781)
Supplement: Supplementary file 1 [file polymers-14-03781-s001.zip › polymers-1907097-supplementary.pdf]

Supporting Information

Trimetallic nanoalloy of NiFeCo embedded in phosphidated nitrogen doped carbon catalyst for efficient electro-oxidation of kraft lignin

Ana Maria Borges Honorato[a,b], Mohmmad Khalid[a,b], Antonio Aprigio da Silva Curvelo[a], Hamilton Varela[a] Samaneh Shahgaldi[b*]

[a]*São Carlos Institute of Chemistry, University of São Paulo. POBox 780, 13560-970, São Carlos, SP, Brazil.*

[b]*Institut d'Innovations en Écomatériaux, Écoproduits et Écoénergies. Université du Québec à Trois-Rivières, 3351 boul. des Forges. Trois-Rivières (Québec) G8Z 4M3, Canada.*

Corresponding author's email: Samaneh.Shahgaldi@uqtr.ca (SS)

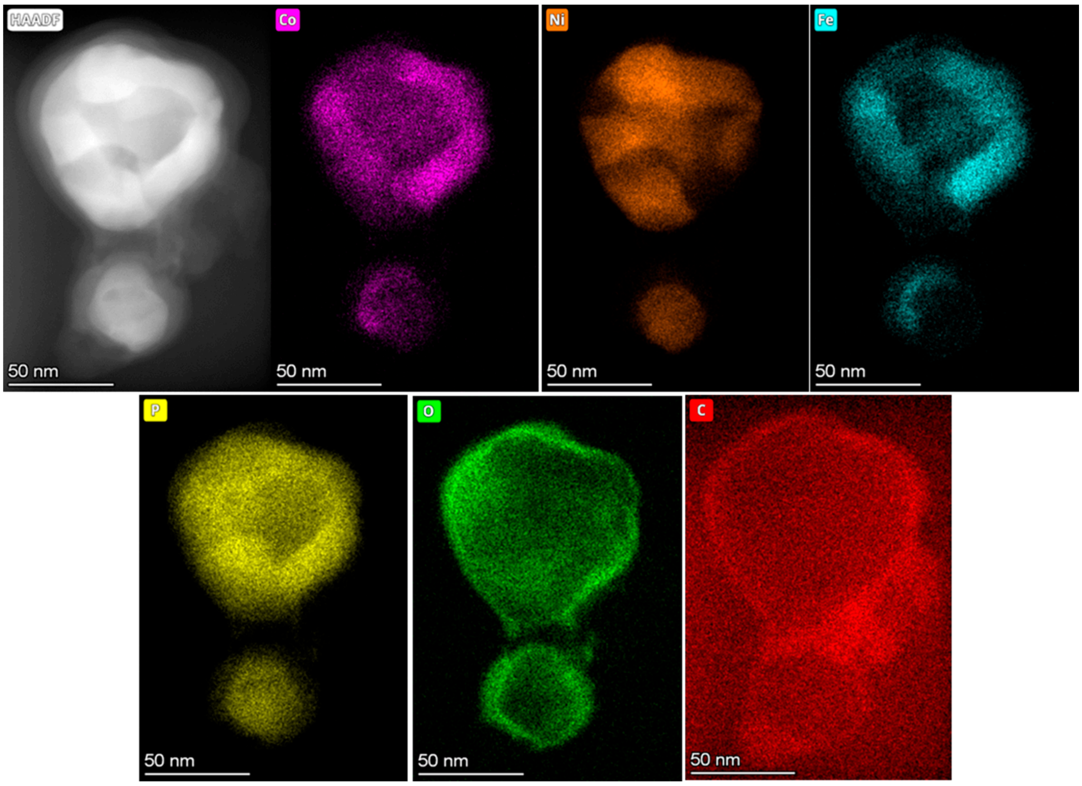

Figure. S1 HAADF-TEM image of NiFeCo nanoalloy and its corresponding elemental mappings Co, Ni, Fe, P, O, and C.

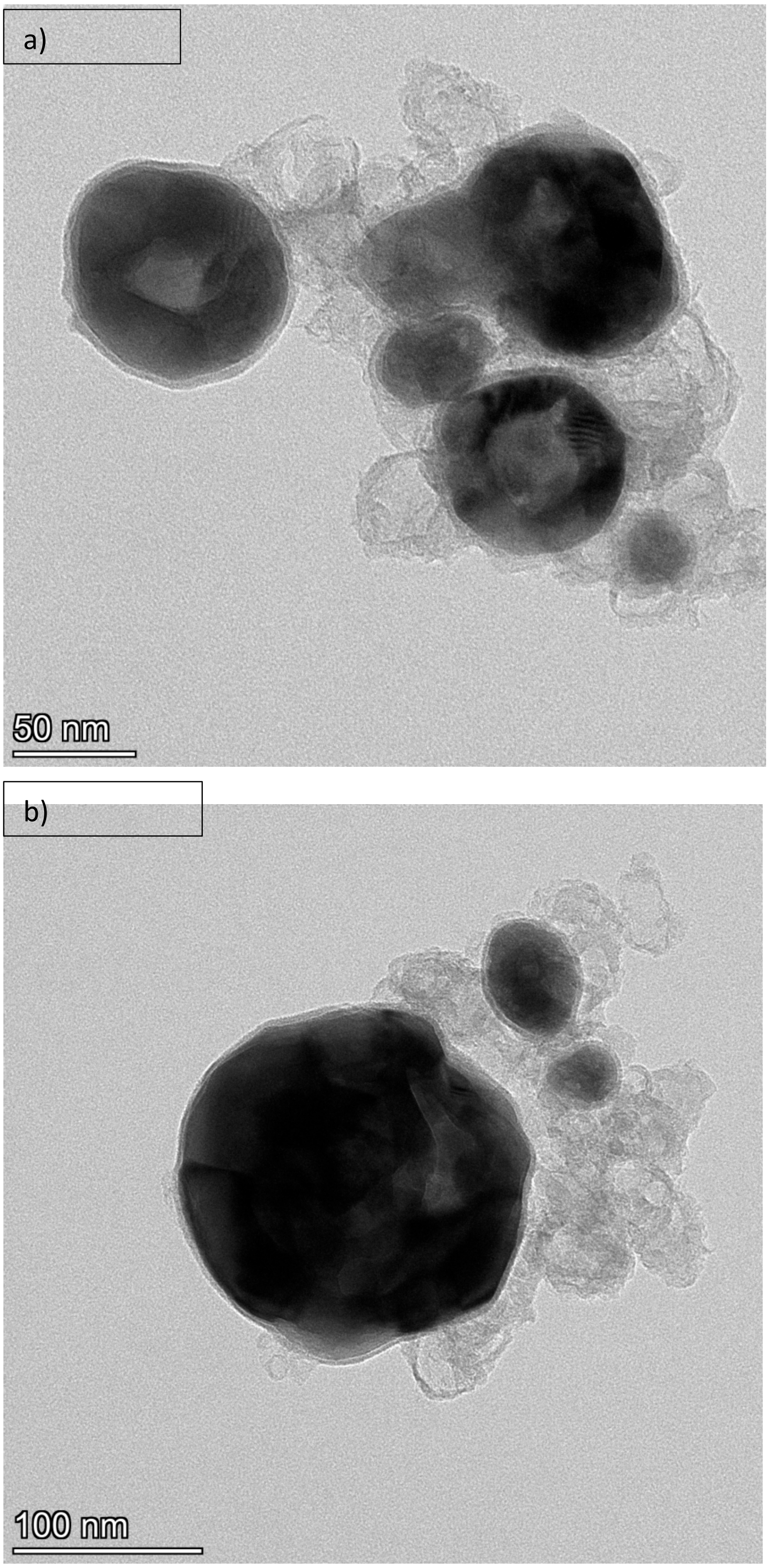

Figure. S2 a,b) TEM image of carbon shell protected trimetallic-nanoalloy.

a)

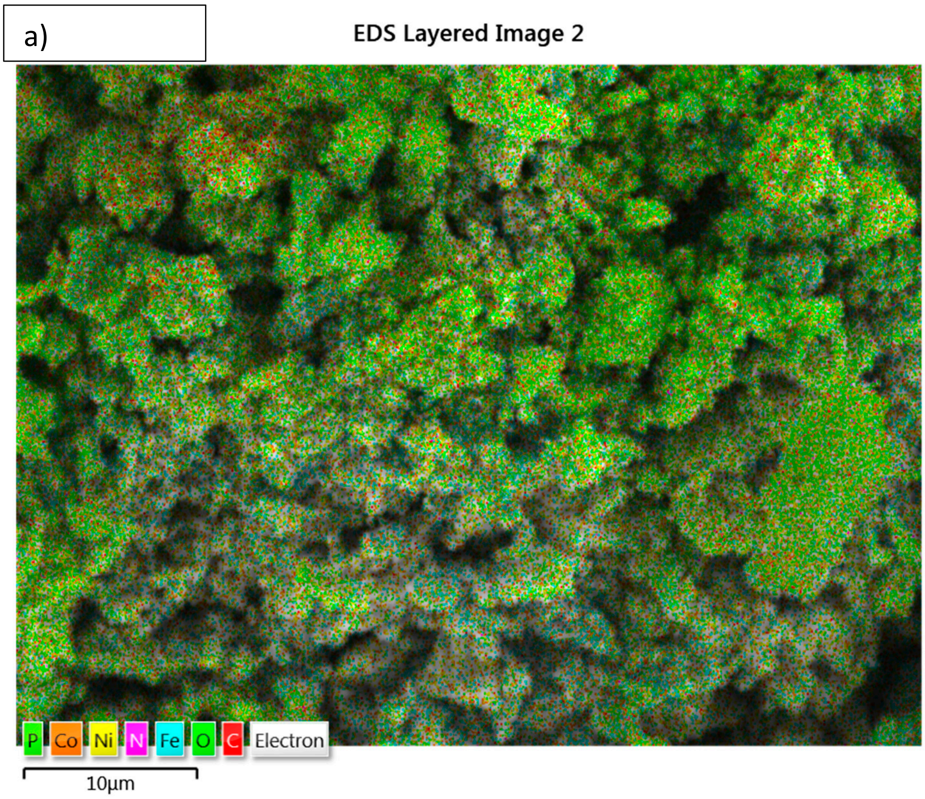

b)

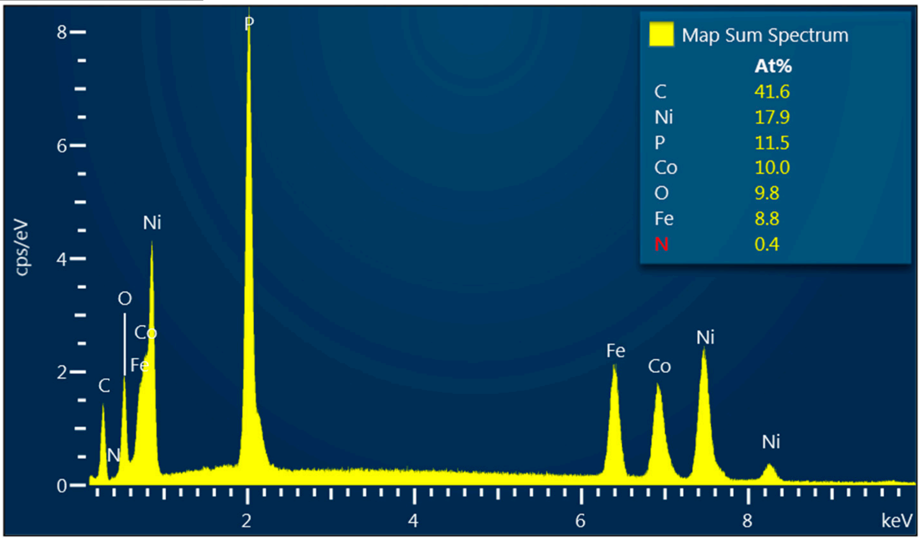

Figure. S3a) EDX mapping for the homogeneous distribution of the elements and **b)** scan line spectrum for the quantification of the elements C (41.6%), Ni (17.9%), P(11.5%), Co(10%), O (9.8%), Fe(8.8%) and N(0.4%).

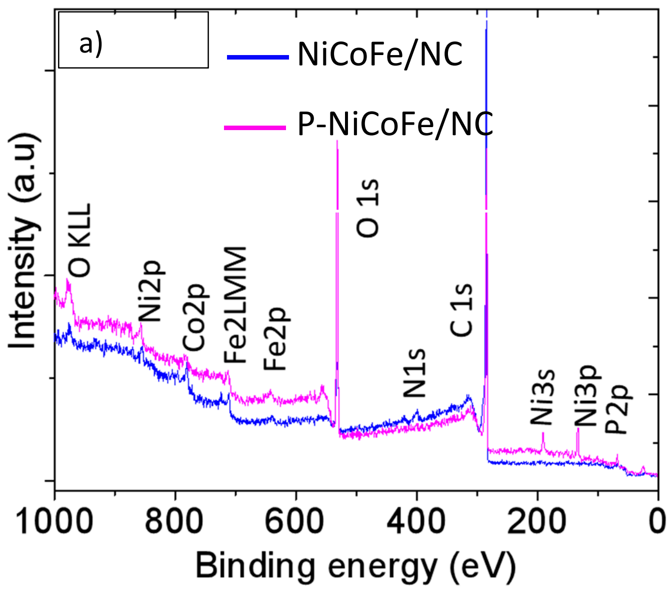

Figure. S4 XPS survey of the samples P-NiFeCo/NC (in pink) and NiFeCo/NC (in blue) showing the peaks of the elements at their specific binding energies.

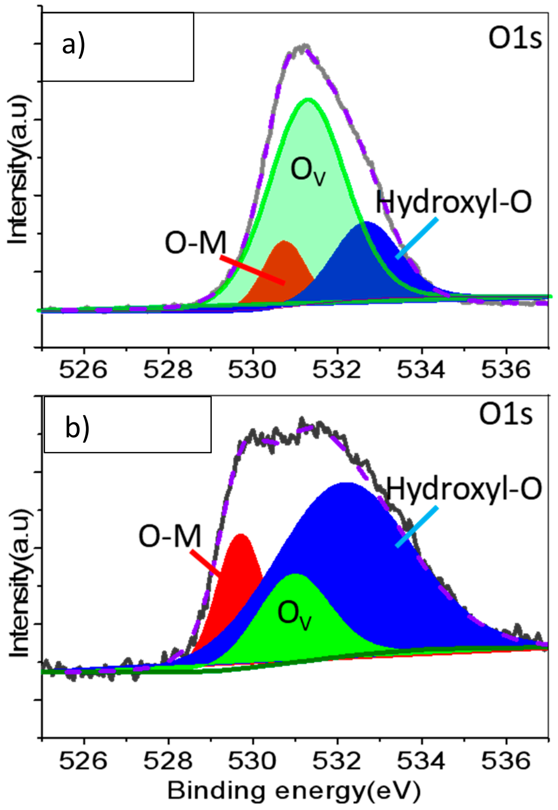

Figure. S5 O1s peaks deconvolution of the sample P-NiFeCo/NC a) and NiFeCo/NC b).

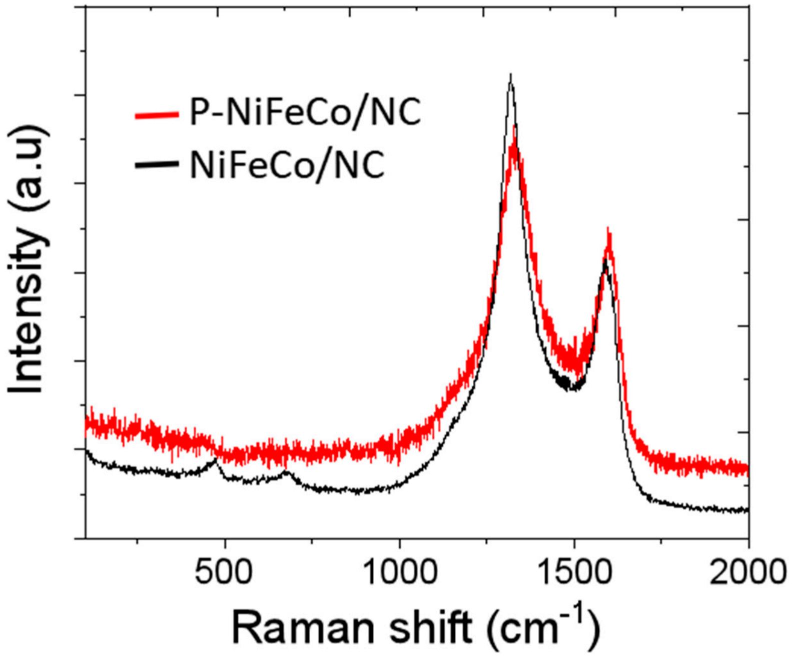

Figure. S6 Raman spectroscopy of the samples P-NiFeCo/NC (in red) and NiFeCo/NC (in black) showing the G and D bands.

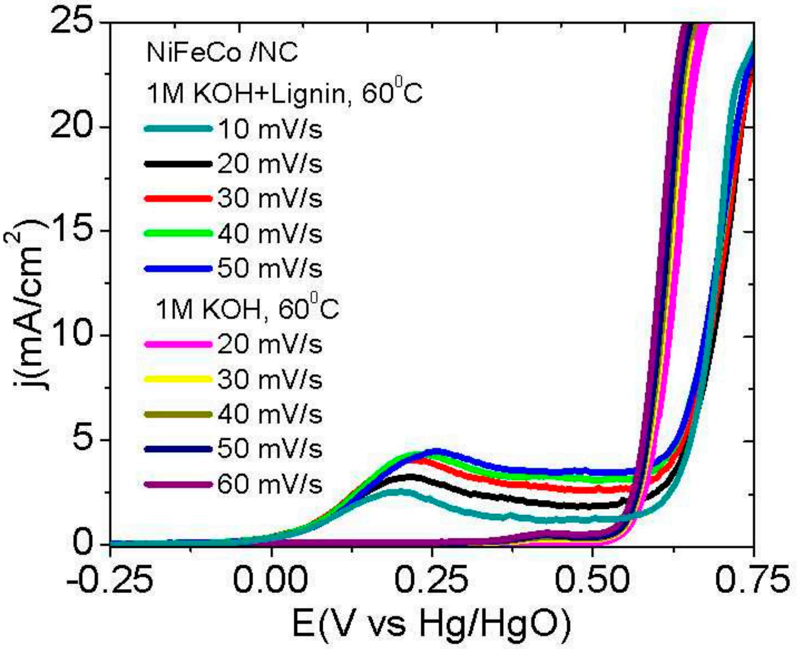

Figure. S7 LSV tests of NiFeCo/NC in 1 MKOH and b) in 1 M KOH + lignin at 60℃.

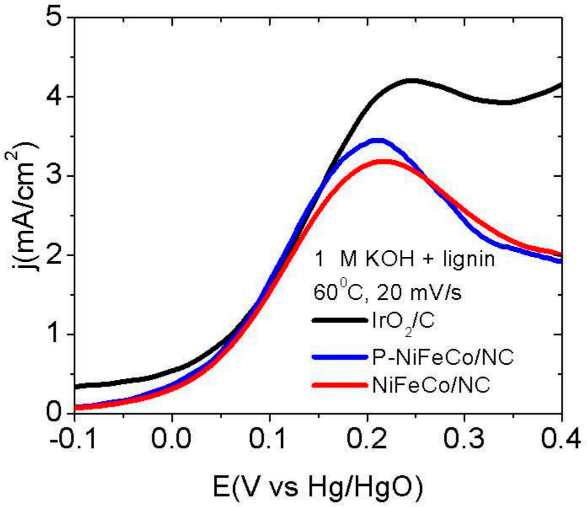

Figure.**S8** LSV tests of IrO_2/C, P-NiFeCo/NC,and NiFeCo/N at 60°C, 20 mV/s in lignin electro-oxidation in 1 M KOH + lignin.

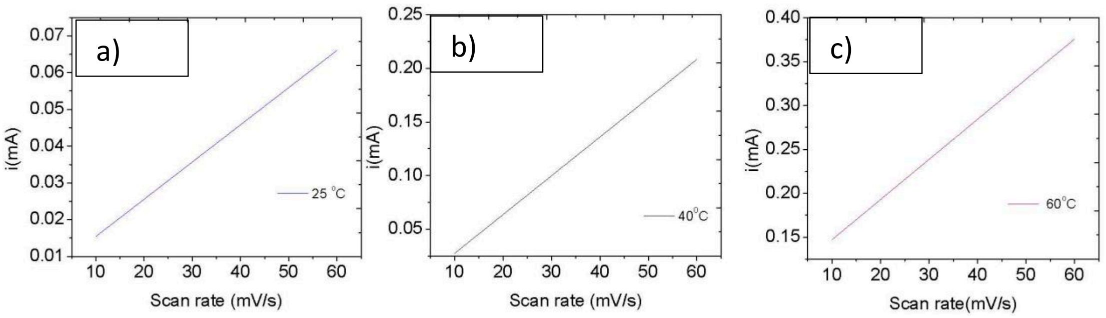

Figure.**S9 a,b,c**) Curves of current at lignin oxidation peaks (mA) versus scan rates from the LSVs taken at 25°C,40°C and 60°C.
